# Supplementary material for: Preparation of Resistant Starch Types III + V with Moderate Amylopullulanase and Its Effects on Bread Properties
Source: Foods. 2024 Apr 19;13(8):1251. doi: 10.3390/foods13081251 (PMC11049056; doi:10.3390/foods13081251)

Supplementary material

Table S1. The resistant starch content of starch samples

Samples	PSn	PSa	PSpa
Resistant starch content (%)	8.2±0.2[a]	8.9±0.2[a]	29.8±0.7[b]

a, b, Different letters are significantly different ($P < 0.05$).

PSn, Native pea starch; PSa, Pea starch treated with autoclaving; PSpa, Pea starch treated with debranching and autoclaving.

Figure S1. SEM images of native and treated pea starches. PSn, Native pea starch; PSa, Pea starch treated with autoclaving; PSpa, Pea starch treated with debranching and autoclaving.

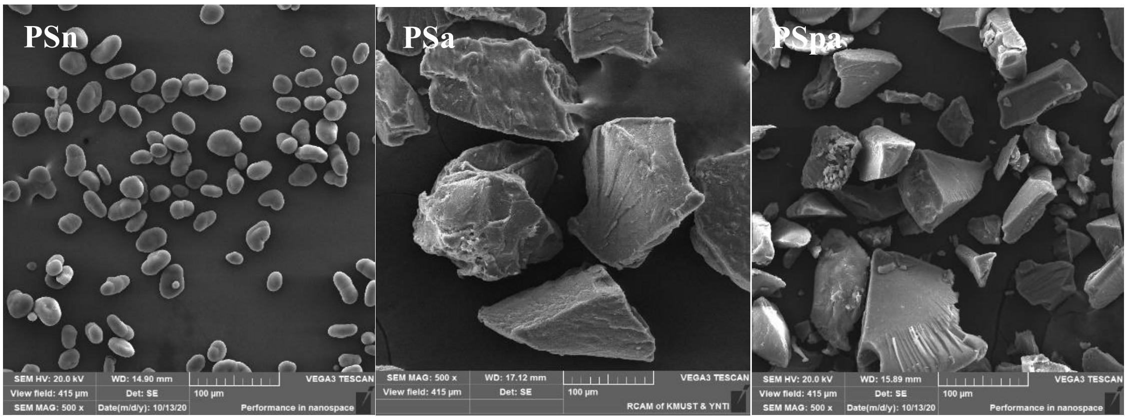

Figure S2. The hardness changes of bread samples during storage time.

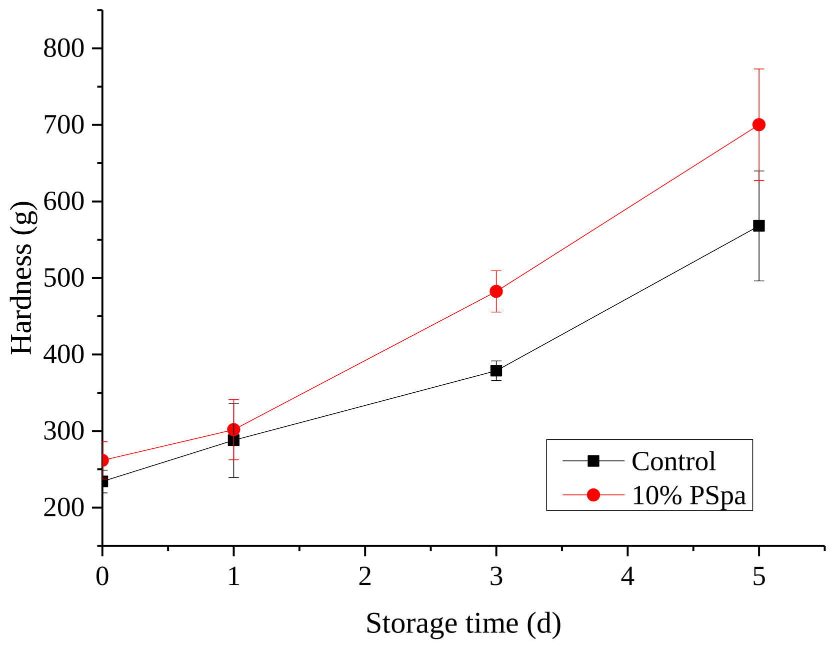

Supplement: Supplementary file 1 [file foods-13-01251-s001.zip › foods-2935316-supplementary.pdf]
